# An Electrochemical Aptasensor for the Detection of Freshwater Cyanobacteria

**DOI:** 10.3390/bios14010028

**Published:** 2024-01-04

**Authors:** Mai-Lan Pham, Somayeh Maghsoomi, Martin Brandl

**Affiliations:** 1 Center for Water and Environmental Sensors, Department for Integrated Sensor Systems, University for Continuing Education Krems, Dr.-Karl-Dorrek-Straße 30, 3500 Krems an der Donau, Austria; somayeh.maghsoomi.taramsari@donau-uni.ac.at (S.M.); martin.brandl@donau-uni.ac.at (M.B.); 2 Institute of Specific Prophylaxis and Tropical Medicine, Medical University of Vienna, Kinderspitalgasse 15, 1090 Vienna, Austria

**Keywords:** *Aphanizomenon*, aptamer, electrochemical, biosensor, aptasensor

## Abstract

*Aphanizomenon* is a genus of cyanobacteria that is filamentous and nitrogen-fixing and inhabits aquatic environments. This genus is known as one of the major producers of cyanotoxins that can affect water quality after the bloom period. In this study, an electrochemical aptasensor is demonstrated using a specific aptamer to detect *Aphanizomenon* sp. ULC602 for the rapid and sensitive detection of this bacterium. The principal operation of the generated aptasensor is based on the conformational change in the aptamer attached to the electrode surface in the presence of the target bacterium, resulting in a decrease in the current peak, which is measured by square-wave voltammetry (SWV). This aptasensor has a limit of detection (LOD) of OD_750_~0.3, with an extension to OD_750_~1.2 and a sensitivity of 456.8 μA·OD_750_^−1^·cm^−2^ without interference from other cyanobacteria. This is the first aptasensor studied that provides rapid detection to monitor the spread of this bacterium quickly in a targeted manner.

## 1. Introduction

Cyanobacteria are Gram-negative photosynthetic bacteria that can be found in both freshwater and marine environments. They are known as blue-green algae and have abilities to adapt to extreme environmental conditions [1,2]. Along with the bloom formation, which causes the deterioration of the water quality, cyanobacteria also produce cyanotoxins as their secondary metabolites released into the aquatic environment, resulting in the death of aquatic organisms and negative effects on human health [3,4]. *Aphanizomenon* is a genus of cyanobacteria that is nitrogen-fixing and filamentous and is one of the primary toxic bloom-forming cyanobacteria [5,6]. Members of this genus can produce several well-known cyanotoxins, including anatoxin-a and saxitoxin, known as neurotoxin alkaloids, causing poisoning syndrome in shellfish [6]; cylindrospermopsin, a guanidine alkaloid resulting in general cytotoxic effects in mammals [6]; and small amounts of microcystin, which is recognized as a potent hepatotoxin leading to serious damage to living organisms’ livers [5]. It is important to quickly identify this genus and monitor its spread, as these bacteria proliferate these toxins in freshwater and marine environments.

Most conventional methods for the detection of cyanobacteria, in general, are based on enzyme-linked immunosorbent assay (ELISA) and liquid chromatography–mass spectrometry (LC–MS), which allow the determination of cyanotoxins. For example, a novel commercial ELISA kit for the measurement of anabaenopeptins was developed by Roy-Lachapelle et al. to monitor the presence of cyanopeptides produced by freshwater cyanobacteria [7]. Also, Method 546 by the US EPA (United States Environmental Protection Agency) based on ELISA determines total microcystins (MCs) and nodularins (NODs) in finished drinking water and surface water [8]. Van Hassel et al. validated a method to evaluate eight microcystin congeners and nodularin in cyanobacteria- and *Chlorella*-based food supplements based on ultra-high performance liquid chromatography–tandem mass spectrometry [9]. However, these methods are time-consuming and costly [7,10].

Alternatively, biosensors have become attractive tools for rapid and accurate detection not only of these toxins but also of typical photosynthetic pigments produced by cyanobacteria [10,11,12,13]. A phycocyanin probe was employed to control cyanobacteria in freshwater bodies of western France [11]. Wang et al. developed a microfluidic biosensor system to rapidly and accurately distinguish living and dead microalgae via their produced chlorophyll fluorescence [13]. A generation of biosensors, known as aptamer-based biosensors or aptasensors, that use aptamers as alternative bioreceptors instead of antibodies in conventional methods have received much attention for various applications [14,15,16]. Compared with antibodies, aptamers have advantages, including their smaller size, lower cost in production, better stability, and longer shelf-life [17]. Additionally, the selection of aptamers does not require an immune response or animals, which are necessary for the production of antibodies [18]. Aptamers are short single-stranded oligonucleotides (DNA or RNA) that have high selectivity and specific binding towards biomolecules due to their adaptive conformation changes in the presence of desired targets [15,16]. Depending on their binding targets and the conditions of their surroundings, aptamers can modify their complex secondary structures from multi-branched loops and junctions to G-quadruplexes [19]. The selection of aptamers is performed by an in vitro process named SELEX (systematic evolution of ligands by exponential enrichment) consisting of several progressive selection rounds from a DNA/RNA library of random sequences [14,15,16,20,21]. Notably, the cell-SELEX process, which consists of several selection rounds to eliminate sequences binding to non-target microorganisms, is exploited to screen aptamers for whole cells and has been applied to select highly specific aptamers for the detection of food-borne and water-borne pathogens [16].

Electrochemical biosensors become potential candidates for diagnosis and environmental monitoring due to their low cost, rapid detection, and ease of manipulation [17,22]. These biosensors allow us to measure the secreted electroactive metabolites and exogenous electroactive compounds of microorganisms (e.g., pyocyanin from *Pseudomonas aeruginosa* [23]), which results in indirectly identifying the presence of target microbes. Furthermore, they can detect microorganisms directly, owing to specific interactions between microbes and a biorecognition probe on the working electrode [22]. Owing to the advantages of aptamers and electrochemical biosensors, various electrochemical aptasensors have been developed for the detection and monitoring of different targets, including whole-cell microbes, proteins, nucleic acids, etc. [24,25,26,27,28,29]. For instance, by exploiting a *Salmonella*-specific aptamer, Ma et al. created an electrochemical biosensor for the detection of *Salmonella* with a low limit of detection of 3 CFU/mL [30]. In another study, a label-free electrochemical aptasensor was developed using single-wall carbon nanotube (SWCNT)-modified screen-printed electrodes to control the presence of *Escherichia coli* O157:H7 in tap water [26].

In this study, we employed a specific aptamer for a freshwater cyanobacterium, *Aphanizomenon* sp. ULC602, selected by a cell-SELEX process, to develop an electrochemical aptasensor to detect the desired bacterium in water. Here, the aptamer was modified with an alkanethiol group at the 5′ end for immobilization on the gold surface of screen-printed gold electrodes (Au-SPEs) (Dropsens) via self-assembly monolayer (SAM) formation and labeled with a methylene blue redox probe at the 3′ end for electrochemical detection by square-wave voltammetry (SWV) measurement.

## 2. Materials and Methods

### 2.1. Bacterial Cultures and Chemicals

*Aphanizomenon* sp. ULC602, *Anabaena* sp. ULC080 and *Microcystis* sp. ULC 641 was obtained from a Belgian coordinated collection of microorganisms (BCCM, University of Liège, Cyanobacteria collection-ULC, Liège, Belgium) [5]. *Aphanizomenon* sp. ULC602 was grown in a 50% dilution of BG110 medium, which was prepared following the manufacturer’s guideline at laboratory room temperature (RT), 23 ± 1 °C, under continuous lighting. *Anabaena* sp. ULC080 was grown in BG110 medium (Table S1), which lacks a nitrate source, at 18 °C under continuous lighting. *Microcystis* sp. ULC641 was cultivated in standard BG11 medium prepared following the manufacturer’s guidelines (Table S2) at RT under continuous lighting. *E. coli* ATCC11330 from cultures collection of UWK (University for Continuing Education Krems) was grown in LB (Luria Broth) medium at 37 °C and 150 rpm. Chemicals for the preparation of culture media were purchased from CarlRoth (Vienna, Austria). Tris buffer, TCEP (tris-(2-carboxyethyl) phosphine hydrochloride), and 6-mercapto-1-hexanol (MCH) were purchased from Sigma-Aldrich (St. Louis, MO, USA). NaH_2_PO_4_, Na_2_HPO_4_, NaCl, MgCl_2_, and PBS (phosphate-buffered saline) were supplied by CarlRoth (Vienna, Austria).

#### ssDNA Library and Aptamer Sequences

A specific aptamer for *Aphanizomenon* sp. ULC602, so-called APS9, was obtained from a random ssDNA library by a 12-rounds-cell-SELEX process (Supplementary Data). The predicted secondary structure with the free energy ΔG of the aptamer was analyzed by the Unafold web server with the following conditions: at 25 °C, 157 mM Na^+^ and 0 mM Mg^2+^, pH 7.4 (Figure S1). The selected aptamer containing 86-nucleotides was then alkanethiol-modified at the 5′-end and labeled with a redox tag Methylene blue (MB) at the 3′-end (Integrated DNA Technologies, Leuven, Belgium) for assembling the aptasensor with the following sequence: 5′-Thiol-C6-TAGGGAAGAGAAGGACATATGATCTTAAGCTCGACTCTGGAGTACCCTAGATGCTACTAGGGCTTGACTAGTACATGACCACTTGA-MB-3′.

### 2.2. Experiment Design

#### 2.2.1. Preparation of Modified Electrodes

The disposable screen-printed gold electrodes (Au-SPEs) (DS 220AT, Dropsens) were used in this study consisting of a working electrode (WE) and an auxiliary electrode (CE) made from gold with a diameter of WE of 4 mm and a reference electrode (RE) made from silver. Before immobilizing on the electrode, 1 µM of thiol-modified aptamer APS9 was reduced by 100 µM TCEP in 50 µL of low-salt 10 mM phosphate buffer (1.9 mM NaH_2_PO_4_, 8.1 mM Na_2_HPO_4_) adding 1.9 mM NaCl and 0.5 mM MgCl_2_ [31] for 2 h at laboratory RT (23 ± 1 °C) in the dark. The reduced aptamer was denatured at 95 °C for 5 min and cooled on ice for 5 min to obtain its stable conformation. Subsequently, 10 µL of the treated aptamer was immobilized on the WE surface for 16 h at RT (23 ± 1 °C) in the dark in a hydrated chamber [31,32]. Afterward, the electrode was washed with deionized water to remove unbound aptamers and then backfilled with 2 mM MCH for 2 h at RT in the dark. Finally, the electrode was rinsed with deionized water and could be used immediately or stored at 4 °C in 10 mM Tris buffer, pH 7.0.

#### 2.2.2. Electrochemical Characterization and Measurement

The immobilization of the aptamer was confirmed by scanning electron microscopy (SEM) using a FlexSEM 1000 instrument (Hitachi, Düsseldorf, Germany) at an acceleration voltage of 20 kV and characterized by cyclic voltammetry using Gamry Reference 600+ potentiostat (Gamry instruments, Warminster, PA, USA) with scanning ranges from −0.7 to +0.5 V, scan rate 50 mV s^−1^. The SWV measurements were performed with a scanning range from −0.5 to +0.5 V at a frequency of 60 Hz, step size of 4 mV, and an amplitude of 40 mV. All measurements were performed at laboratory RT (23 ± 1 °C). The decrease of the peak currents (ΔI) was defined as the different currents in the absence and presence of the target bacterium measured by SWV and was calculated by:

(1) $$∆I=I_{0}-I$$

- ΔΙ: Decrease of peak current
- I_0_: Current measured in the absence of the bacterium by SWV
- I: Current measured in the presence of the bacterium by SWV

The signal loss is the percentage of decrease of peak current compared to the current measured in the absence and presence of the target bacterium and was calculated as:

(2) $$Signalloss\left[\%\right]=\frac{∆I}{I_{0}}\cdot 100\%$$

##### Stability of the Modified Electrode

The modified SPEs were stored at 4 °C in 10 mM Tris buffer, pH 7.0, for several weeks. Every week, three modified electrodes underwent the SWV measurement for a period of 5 weeks. The signal loss in percentage every week was recorded compared to the initial SWV measurement as in Equation (2). The stability of the SWV signal was tested for 2 h in PBS 1X buffer, pH 7.4. The signal loss in percentage was recorded every 15 min and was calculated as in Equation (2) above.

#### 2.2.3. Detection of the Target Bacterium

The target bacterium, *Aphanizomenon* sp. ULC602, was grown in its medium under the standard conditions mentioned above. Bacterial cells were collected at different optical densities (OD) at 750 nm and re-suspended in PBS 1X (137 mM NaCl, 2.7 mM KCl, 10 mM Na_2_HPO_4_, and 1.8 mM KH_2_PO_4_), adding 0.05% (*v*/*v*) Tween 20. A newly modified Au-SPE was incubated with every concentration of *Aphanizomenon* sp. ULC602, based on the collected OD_750_ at 25 °C for 1 h. After binding, the electrodes were rinsed with PBS 1X and deionized water to remove unbound cells. They were measured using SWV signals following the same conditions as described above.

The selectivity of electrodes was performed using mixtures of the target bacterium and control bacteria collected at the same OD_750_ of 0.5, including *Anabaena* sp. ULC080, *Microcystis* sp. ULC641 (BCCM) and *E. coli* ATCC11330. The binding of every bacterial mixture was carried out on three different modified SPEs.

#### 2.2.4. Statistical Analysis

All experiments and measurements were conducted in triplicate. Data analyses were performed statistically using Sigma Plot ver 11.0 software (Systat Software Inc., San Jose, CA, USA). The data are expressed as the mean ± standard deviation (SD) when appropriate.

## 3. Results and Discussion

**Figure 1:**
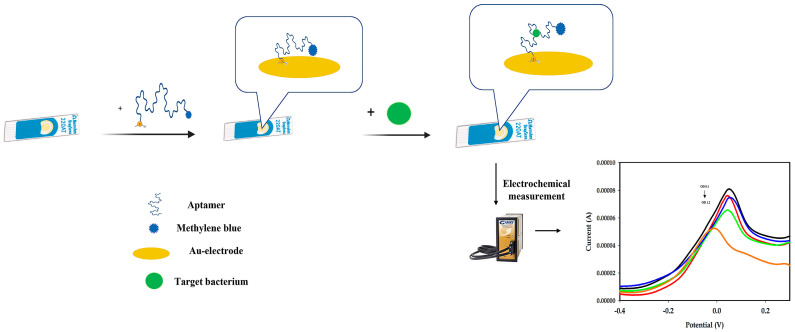
Schematic illustration of the aptasensor and the working principle using an Au-SPE (Dropsens) for the detection of *Aphanizomenon* sp. ULC602 (created by BioRender.com). (1) The fabrication of the Au-SPE: the thiolated aptamer was covalently bound on the gold electrode via a gold-thiol SAM, bringing the MB tag close to the gold surface. (2) Detection of *Aphanizomenon* sp. ULC602: the fabricated electrode was incubated with the target bacterium at 25 °C for 1 h. The aptamer recognized the target bacterium and opened its loop structure to bind the target, leading to the move of the MB probe further away from the electrode’s surface. (3) The electrochemical signal of the MB probe was measured.

The strategy for the construction and detection of *Aphanizomenon* sp. ULC602 is illustrated in Figure 1.

### 3.1. Modification of Au-SPEs

To immobilize the aptamer on the gold electrode, the APS9 was modified with an alkanethiol group (Thiol-C6) tagged at the 5′ end of the oligos and treated with TCEP to form a SAM of the aptamer on the gold surface (Figure 1, step 1). The thiol-SAMs were demonstrated to be highly stable due to the strength of the S-Au bond and the van der Waals interactions of the hydrocarbon chain [33]. The utilization of thiol-SAMs on gold material as building blocks for sensors and biosensors has been mentioned in many studies [33,34]. It should be noted that the alkyl chain length affects the formation of SAMs [34]. Here, the APS9 was linked to the thiol group via a C6 linker to ensure sufficient space for the folding of the aptamer as well as to enable backfilling with MCH, which is used to overcome the non-specifically adsorbed part of the thiolated aptamer [34].

**Figure 2:**
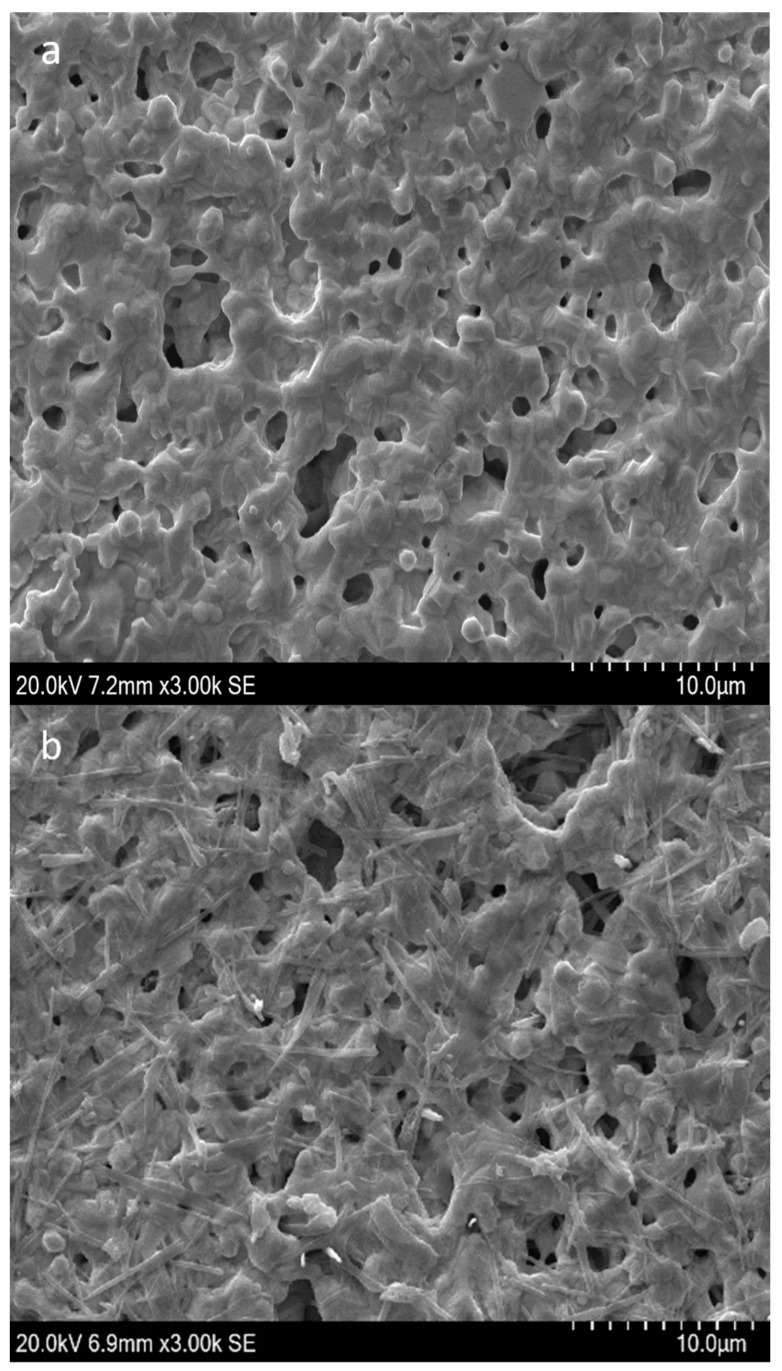

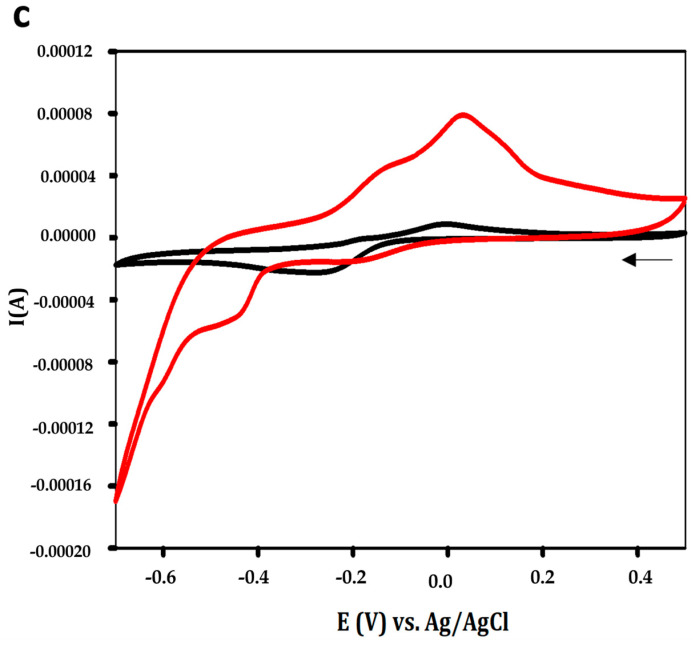
SEM micrograph of (**a**) the bare Au-SPEs; (**b**) the modified Au-SPEs by assembly of APS9 on the gold electrode; (**c**) cyclic voltammogram of the bare Au-SPEs (black) and the modified Au-SPEs (red) using PBS 1X, pH 7.4 as the working buffer and with scan ranges from −0.7 to +0.5 V at a scan rate of 50 mV·s^−1^. The arrow indicates the scanning direction.

The APS9 was simultaneously treated before the immobilization to adopt its stable conformation with a stem-loops structure, which was predicted by the Unafold webserver (Figure S1) for the binding to *Aphanizomenon* sp. ULC602. The successful fabrication of the created sensor was observed by SEM at a scale of 10 µm with high magnification of 3000X and by CV (cyclic voltammetry) measurements, as shown in Figure 2. Compared to the bare electrode (Figure 2a), the electrode’s surface of the modified electrode became rougher with the appearance of the tubular structure of the aptamer, verifying the immobilization of the aptamer on the surface of the Au-SPEs [35,36]. Figure 2c illustrates the difference in shapes of the CV curves at a low scan rate of 50 mV·s^−1^ with a scanning range from −0.7 to +0.5 V before and after the fabrication of the electrodes, which confirmed the successful immobilization of the APS9 to the gold electrode. Due to the immobilization of the APS9 on the SPEs, the redox probe, MB, which is tagged at the 3′ end of the APS9, is brought closer to the surface of the electrode, resulting in the production of an electrochemical signal from MB (red line, Figure 2c). Also, in other studies, MB was chosen as a redox mediator for the development of aptasensors [26,31,32,37,38]. Several strategies employing MB as a redox mediator in aptasensors as well as DNA sensors have been applied: the MB can either electrostatically interact with the DNA or intercalate in the DNA double helix between the G-C sequence [39]. It also can covalently bind to the end of the ssDNA [39,40]. Lubin et al. indicated that the electrochemical signal generated on the gold electrode by employing the MB-tagged ssDNA, similar to our study, was connected to a hybridization-linked conformational change [40]. In Figure 2c, a reductive peak at −0.48 V and two oxidative peaks for MB oxidation (red lines) at −0.04 V and −0.11 V were found at the modified electrodes in PBS buffer, pH 7.4. The possible appearance of two oxidative peaks of the leucomethylene blue due to the adsorption of the MB products has been reported in several studies [41,42,43,44,45]. It could be due to the protonation equilibrium of intermediates, which are hard to oxidize [45,46]. It is supposed that the change in hydrogen bonding interactions of the ssDNA and MB might impact the protonation reaction of the intermediates [45]. It should be mentioned that this observation depends on the type of electrode, working buffer, and pH [41,46]. In addition, a redox wave was detected at the bare electrode (black line), which is believed to be from the electrochemical signal of gold using the PBS buffer, pH 7.4 as the electrolyte [47,48].

### 3.2. Characterization of the Aptasensor

**Figure 3:**
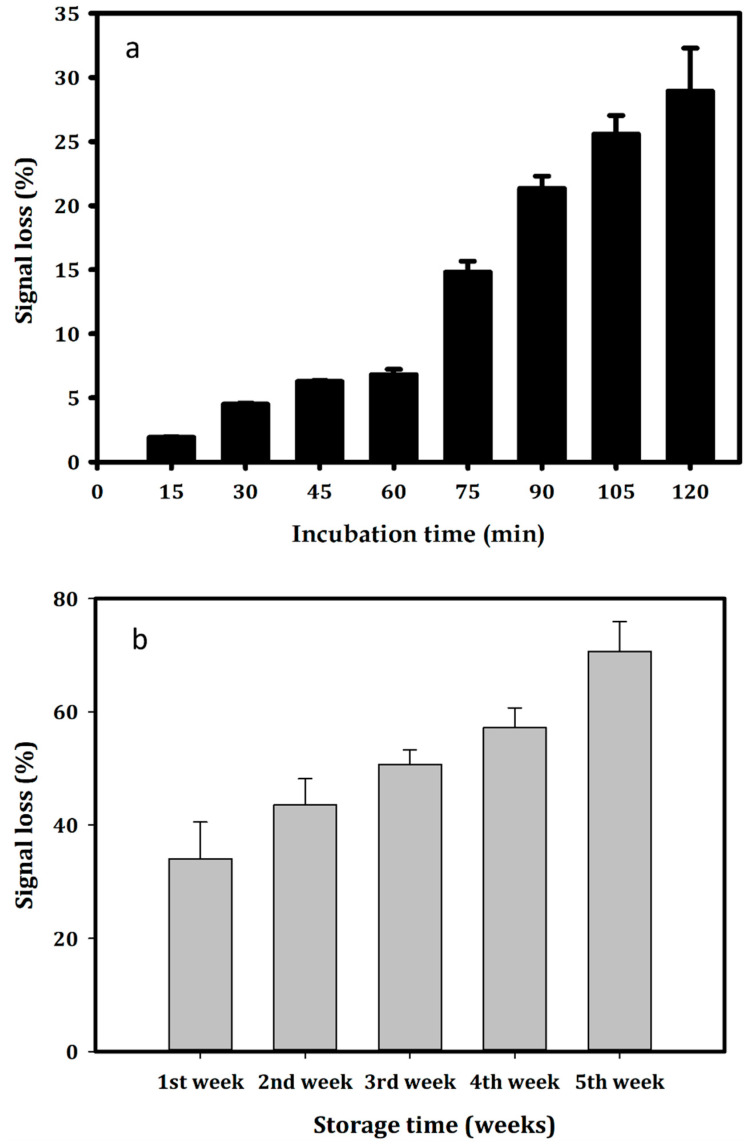
Stability of (**a**) the faradaic current of the aptasensor in PBS 1X buffer, pH 7.4 for 2 h; (**b**) the aptasensor stored at 4 °C in Tris buffer, pH 7.0. All measurements were performed by SWV.

The stability of the faradaic current for the modified electrodes was determined by SWV in PBS 1X buffer, pH 7.4 for 2 h, which is expressed in Figure 3a (One-way ANOVA: n = 26; F = 204.374; *p* < 0.001). The obtained results showed that the electrochemical signal of the generated aptasensor was stable in the first hour, with a signal loss of 2% after 15 min (Holm–Sidak: *t* = 1.808, *p* = 0.087) and 4.4% after 30 min (Holm–Sidak: *t* = 4.236; *p* < 0.001). After one hour of incubation, the signal loss was recorded as 6.8%, compared to the initial signal (Holm–Sidak: *t* = 6.407; *p* < 0.001). The electrochemical signal decreased to 70% compared to the initial signal, which is equivalent to a signal loss of 30% (Holm–Sidak: *t* = 27.165; *p* < 0.001). The formed stem-loops secondary structure of the APS9 (Figure S1) could be one of the reasons leading to the decrease in the signal. The signal collected from the electrochemical stem-loops DNA sensor was expected to possibly reduce by 30–70% at RT (depending on probe density) [49,50].

Notably, the constructed aptasensor reduced the SWV peak current to 57% after 4 weeks (Holma–Sidak: *t* = 16.307; *p* < 0.001) and 70% after 5 weeks storage in Tris buffer, pH 7.0, at 4 °C (Holma–Sidak: *t* = 20.150; *p* < 0.001) (Figure 3b). The long-term stability of this aptasensor was not as good as those in other studies. For instance, the aptasensor generated on the same Au-SPEs for detecting estrogen receptor alpha in breast carcinomas could maintain up to 95% of the signal after 60 days of storage at 4 °C [28]. The electrochemical signal of the MUC1 aptasensor was shown to be stable for 2 h with a maximum signal loss value of 10% [48].

### 3.3. Detection of Aphanizomenon sp. ULC602

**Figure 4:**
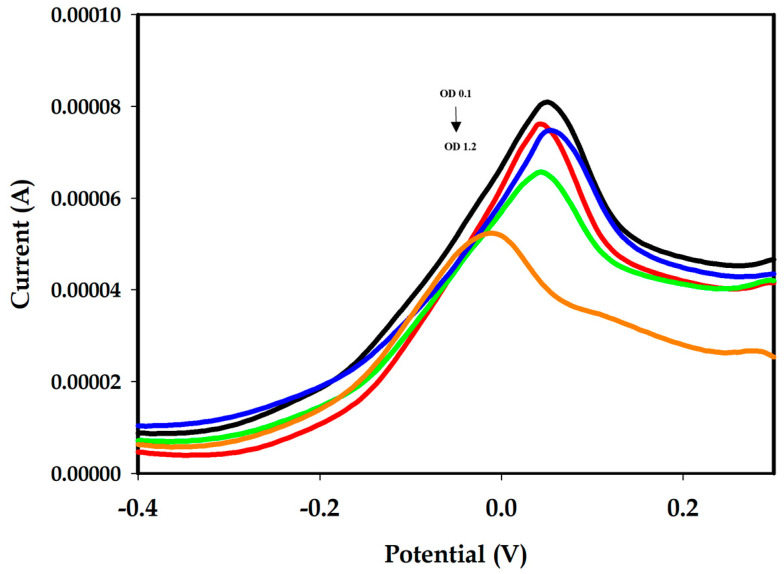
SWV of the modified Au-SPEs incubated with different amounts of *Aphanizomenon* sp. ULC602, defined by OD_750_ in PBS 1X, pH 7.4. The current peak decreased with the increase in the number of bacterial cells (OD_750_~0.1 to 1.2, black to orange). The SWV measurement of each OD_750_ was performed in different disposal-modified Au-SPEs.

Different amounts of *Aphanizomenon* sp. ULC602 (from OD_750_ 0 to 1.2), described in terms of OD at the wavelength of 750 nm (OD_750_) by UV–vis spectrophotometry, were incubated on different disposal-modified SPEs at 25 °C for 1 h. The experiments were triplicated. The SWV measured in the potential range of −0.5 V to +0.5 V is represented in Figure 4. The SWV signal obtained from the working buffer (PBS 1X, pH 7.4) is considered to be blank, containing no bacterial cells. A decrease in the peak current was observed with the increase in the number of bacterial cells. The obtained results illustrated the working principle of this aptasensor, which is based on the induction of DNA folding. Briefly, in the absence of the target bacterium, the APS9 was in its stable conformation with a stem-loops structure, which brought the MB tag at the 3′-end close to the surface of the electrode, resulting in a high electrochemical signal by the MB. On the other hand, the presence of the target bacterium induced the conformational change in the APS9 for biorecognition. As a result, the loop structure of APS9 was opened to bind to the target, increasing the distance between the MB tag and the surface of the electrode, which decreased the electron transfer, leading to a decrease in the SWV signal (Figure 1, step 2 and step 3). The “signal-off” mechanism observed from this aptasensor has also been described for other targets in previous studies [26,48,51]. Notably, the potential was moved slightly negatively when the amount of the target bacterium increased (Figure 4). The decrease of the electron transfer between the MB tag and the surface of the electrode in the high concentration of bacterial cells may cause it. In addition, the characteristic results also showed that the proposed aptasensor was unstable compared to the previously studied sensor, which can result in this negative movement.

**Figure 5:**
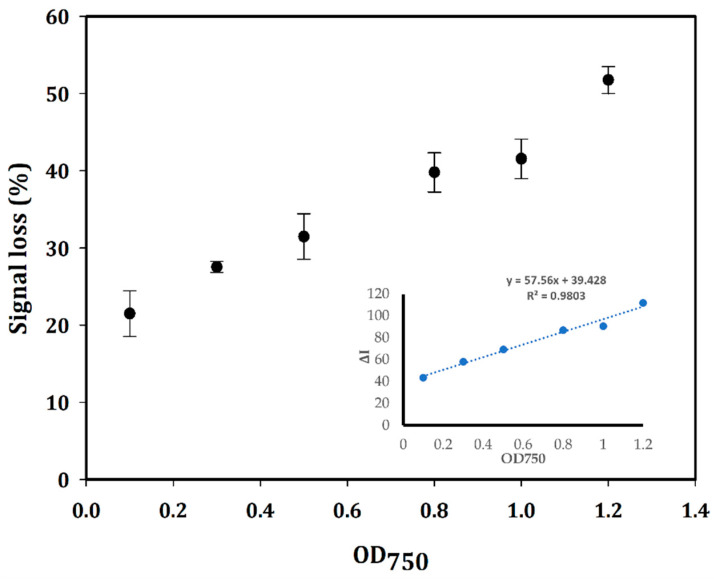
Plot of signal loss in percentage (%) for different amounts of bacterial cells defined as OD_750_ and the linear calibration curve of the aptasensor plotted by the decrease of peak current (ΔI) vs. OD_750_ with R^2^ = 0.9803. The SWV measurements were conducted on three different modified Au-SPEs. The mean and SD values are based on an average of measurements on three different modified SPEs.

The signal loss in percentage, which was calculated as Equation (2) and plotted with different amounts of bacterial cells as OD_750_, is illustrated in Figure 5. The ΔI value, as the current difference between the absence (the blank containing no bacterial cells) and presence of the bacterial cells, was calculated according to Equation (1) and plotted vs the OD_750_ (Figure 5) to create a linear calibration curve with the equation: y = 57.56x + 39.428 with R^2^ = 0.9803. The sensitivity of this aptasensor was determined according to the following formula: S = m/A, where m is the slope of the calibration curve, and A is the area of the WE [35]. The limit of detection (LOD) is defined as the lowest concentration of bacterial cells in the sample at which the electrochemical signal can be measured by the created sensor with an acceptable threshold of 3. This value is calculated based on the SD of the response and the slope of the calibration curve as the formula: LOD = 3·(σ/m), where σ is the SD of the response and m is the slope of the calibration curve [35]. In this study, the concentration of bacterial cells was defined as the optical density, which was measured at 750 nm to avoid the absorption interference with natural pigments of cyanobacteria [52,53]. From the calibration curve, the sensitivity and LOD values were calculated to be 456.8 µA·OD_750_^−1^·cm^−2^ and OD_750_ of 0.3, respectively, on an electrode with 0.126 cm^2^ surface area. The electrochemical signal of the designed aptasensor lost 6.8% after 1 h at RT. It could be due to the degeneration in the performance of the RE after continuous measurements in the same SPEs [54,55,56]. Therefore, each calibration point was measured in three different modified SPEs. The reproducibility of the created aptasensor on three different modified SPEs with OD_750_~0.5 gave an RSD value of 6.521%. In addition, the reproducibility of the aptasensor with OD_750_~1 gave an RSD value of 5.32%. However, it is not a considerable matter because of the disposable (one-time use) property of the SPEs.

**Figure 6:**
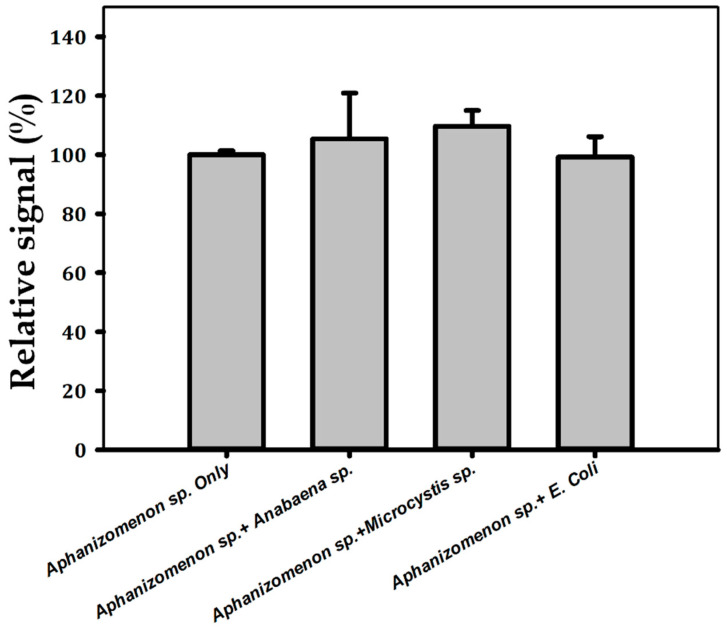
Selectivity of the aptasensor evaluated with interfering species in the mixtures of *Aphanizomenon* sp. ULC602 with *Anabaena* sp. ULC080, *Microcystis* sp. ULC641 and *E. coli* ATCC11330. All bacteria were obtained at OD_750_~0.5. The signal obtained from *Aphanizomenon* sp. ULC602 only is considered to be 100%.

The selectivity of the constructed aptasensor was determined by analyzing the influences of interfering species of mixtures containing *Aphanizomenon* sp. ULC602 and other bacteria, as shown in Figure 6. Two other cyanobacteria, *Anabaena* sp. ULC080, representing filamentous cyanobacteria, *Microcystis* sp. ULC641, representing unicellular cyanobacteria, and *E. coli* ATCC11330, representing Gram-negative bacteria mixed with the target bacterium, were tested. It was found that there was no significant effect on the measurement current when compared with the sample containing only *Aphanizomenon* sp. ULC 602, whose signal loss was considered 100%, confirming the selectivity of the sensor towards the target bacterium. One-way ANOVA statistical analysis confirmed the differences between the groups; the means were not statistically significant, with a *p*-value of 0.477.

## 4. Conclusions and Future Perspectives

This study successfully offers a novel electrochemical aptasensor designed on gold-SPEs for the highly sensitive and rapid detection of filamentous cyanobacteria in water. The specific aptamer for *Aphanizomenon* sp. ULC602, APS9, could be covalently immobilized on the surface of the gold electrode via thiol-Au bonding and labeled with MB as a redox tag. The created aptasensor works based on the induction of the aptamer folding mechanism when detecting the target object, which is then converted into a “signal-off” switch measured by SWV. The sensor showed high selectivity towards the target bacterium as well as high sensitivity with a detection limit of OD_750_ = 0.3. Indeed, enumeration of filamentous cyanobacteria is a challenge because of their complex cell morphology. This means that direct counting of individual cells is difficult to achieve. The estimation of the bacterial cell concentration based on turbidity (OD) is widely used to monitor the growth of bacteria but can create some errors during the measurement because it is not suitable for filamentous photoautotrophs. Therefore, to further improve and validate the constructed aptasensor, a suitable counting method for estimating cell concentration should be defined. In addition, from the obtained results, the stability of this sensor should be improved by optimizing the immobilization buffer, the working electrolyte, pH, and temperature conditions. Alternatively, other materials for the construction of the electrode can be used to improve the performance of the sensor. In addition, the sensor should be tested with different bacterial strains and real water samples to evaluate its suitability for monitoring the presence of the desired cyanobacterium. Although the proposed sensor should be further optimized, this is the first aptasensor that provides an alternative for rapid and specific monitoring of the spread of cyanobacteria.

## Data Availability

The data from this study are available under the “TraceSense” project with the ID: WST3-F-5030664/027-2020.

## Supplementary Materials

The following supporting information can be downloaded at: https://www.mdpi.com/article/10.3390/bios14010028/s1, Oligonucleotide library and SELEX process [57]; Figure S1: Predicted secondary structure of the aptamer APS9 by Unafold web server; Table S1: Component of BG110 (Std) in 1 L (BCCM, University of Liège, Cyanobacteria collection-ULC, Liège, Belgium); Table S2: Component of BG11 medium in 1 L (BCCM, University of Liège, Cyanobacteria collection-ULC, Liège, Belgium). Table S3: Sequences of aptamer candidates analyzed by sequencing. Constant regions are in italics.

## Abbreviations

Au: gold; CV: cyclic voltammetry; MB: methylene blue; OD: optical density; PBS: phosphate-buffered saline; RT: room temperature; SPEs: screen-printed electrodes; RSD: relative standard deviation; SAM(s): self-assembly monolayer(s); SWV: square-wave voltammetry.
