# Supplementary material for: An Electrochemical Aptasensor for the Detection of Freshwater Cyanobacteria
Source: Biosensors (Basel). 2024 Jan 4;14(1):28. doi: 10.3390/bios14010028 (PMC10813013; doi:10.3390/bios14010028)
Supplement: Supplementary file 1 [file biosensors-14-00028-s001.zip › biosensors-2688493-supplementary.pdf]

Supplementary materials

1. Oligonucleotide library

A random 86-Nucleotides ssDNA library was synthesized with the following sequence: 5'-TAG GGA AGA GAA GGA CAT ATG AT-N_{40}-TTG ACT AGT ACA TGA CCA CTT GA-3', where the central N_{40} represents random oligonucleotides based on equal incorporation of A, T, G and C at each positions (Microsynth, Vienna, Austria) . The forward primer (5'-TAG GGA AGA GAA GGA CAT ATG AT-3'), unmodified reverse primer (5'-TCA AGT GGT CAT GTA CTA GTC AA-3') and biotinylated reverse primer (5′-biotin-TCA AGT GGT CAT GTA CTA GTC AA-3') were synthesized by Microsynth. The synthetic oligonucleotides were prepared in nuclease-free water to final concentration of 100 µM (Microsynth).

2. Whole cell-SELEX process

The whole cell SELEX process included total twelve rounds and was adapted from Kolm et al. with some modifications. The target bacterium, *Aphanizomenon* sp. ULC 602 was grown in 50% BG 110 medium at laboratory room temperature (23 ± 2 °C) under continuous light and was harvested at OD_{750} ~ 0.5 for binding experiments. In the first round of SELEX, the ssDNA library was prepared in 50 µl of the binding buffer (PBS 1X buffer adding 0.05% (v/v) Tween 20, pH 7.4), heated at 95 °C for 5 min and folded at 21 °C for 30 min. 50 µl of the folded ssDNA pool was incubated with 200 µl of bacterial cell suspension at 25°C for 1 hour, gentle shaking. After the incubation, bacterial cells were collected and washed one time with the binding buffer. The aptamer-binding cells was re-suspended in 50 µl of the nuclease-free water and heated at 95 °C for 10 min to elute the cell-bound ssDNA from the first round. A negative control containing only bacterial cells with no adding ssDNA pool were prepared and run in parallel with the entire procedure of the selections for cross-contamination checking. The entire collected ssDNA of the first round was amplified by preparative PCR. The PCR was prepared in a reaction volume of 25 µl using Q5 HighFidelity Master Mix (New England Biolabs, Germany), 500 nM forward primer, 500 nM biotinylated reverse primer and 1 µl of the cell-bound ssDNA. The PCR reactions were conducted by Thermocycler Multigene Optimax (Labnet Int., Inc, USA) following the thermal cycling conditions: an initial step of 30 s at 98°C, followed by 10 s at 98°C, 15 s at 56°C, 15 s at 72°C and a final extension step for 2 min at 72°C. The number of PCR cycles were optimized for every SELEX round to avoid the formation of by-product by a real-time PCR reaction before the preparative PCR. The maximum fluorescence was reached at the cycle 25 of the real-time PCR reaction before the amplification curve and this cycle number was used for preparative PCR.

ssDNA preparation for other rounds

The ssDNA pool for subsequent rounds was prepared from the preparative PCR products of the previous round by alkalin denaturation and affinity purification with Steptavidin coated magnetic beads (New England Biolabs, Germany). Briefly, the PCR products were purified from the reaction mixture using Exo-CIP rapid PCR kit (New England Biolabs, Germany) following the instruction of manufacture. The purified double-stranded PCR product was denatured at 95 °C for 5 min and cooled at 4

°C to separate strands. The denatured DNA was incubated with pre-washed Steptavidin coated magnetic beads in a Tris- binding buffer containing 10 mM Tris-HCl, 1mM EDTA, 2 M NaCl for 25 min at room temperature with rotation. The DNA-bound beads were washed with the Tris- binding buffer, then re-suspended in 100 µl of 0.15 M NaOH and incubated at room temperature for 2 min with rotation. Subsequently, the beads were precipitated, and the supernatant was neutralized by titration with 0.15 M HCl. The eluates containing ss DNA pool were then filtered through a Spin-X centrifugation filter (0.22 µm, Corning, NY) to completely remove all beads. The sizes of purified dsDNA and ssDNA of each round were analyzed on 2% agarose gel stained with SYBR Safe DNA gel Stain (ThermoFisher Scientific).

Subsequent SELEX rounds

The SELEX conditions of subsequent rounds were similar as the first round, except some modifications. The amounts of input ssDNA pool were decreased from 2 nmol to 0.1 nmol in subsequent rounds. The washing cycles were increased to 5 cycles. 1% BSA (Sigma) and 25 $mg.ml^{-1}$ Salmon Sperm DNA were added to the binding buffer. In addition, to exclude the non-specific binding sequences, counter selection was performed by incubating the ssDNA pool with non-target bacteria including *E. coli* ATCC 11330 (OD_{750} ~ 0.5) and *Anabaena* sp. ULC 080 (OD_{750} ~ 0.5) in binding buffer at 25 °C for 1 hour. The supernatant containing unbound ssDNA collected from the counter selection was then incubated with the target bacterium (OD_{750} ~ 0.5).

Quantitative PCR

The total ssDNA of each round in SELEX process was quantified via quantitative PCR (qPCR). qPCR reactions were conducted on LightCycler 96 Real-Time PCR System (Roche, USA) according to the following thermal cycling conditions: 30 s at 95°C, followed by 40 cycles of 15 s at 95°C and 30 s at 60 °C. The total volume of a qPCR mixture is 15 µl containing 1X Lunar Universal qPCR MasterMix (New England Biolabs, Germany), 0.25 µM of each primer (forward primer and unmodified reverse primer), 1 µl of cell-bound ssDNA (in the first round, 5µl of template was used). All reactions were triplicated, including no-template control in each run for contamination checking. A set of 5 standard series dilutions (from 10^3-10^7 ssDNA copies per reaction) was prepared and included in each qPCR run. The quantification of ssDNA was based stand curve created from prepared dilutions. Samples were measured undiluted and diluted 1:10 in nuclease-free water. Samples were assumed to be free of inhibition if concentrations in the dilutions matched.

Cloning and sequencing

The aptamer pool obtained from the 12th round of the SELEX was amplified by PCR using Q5 HighFidelity Master Mix (New England Biolabs, Germany) and unmodified primers. The PCR products was ligated to pMiniT 2.0 vector (New England Biolabs, Germany) following the instruction of the manufacture. The ligated plasmid was then chemically transformed into *E. coli* NEB10β by heatshock as the manufacture's instruction. The transformed cells were spread on LB adding 100 µg/ml Ampicillin agar plates. The plates were incubated at 37 °C overnight. After the incubation, 20 random single colonies were chosen for colony PCR using OneTaq Master Mix with Standard Buffer (New England Biolabs, Germany), 200 nM forward primer, 200 nM unmodified reverse primer,

1 µl of total genome isolated from colonies in a reaction of 25 µl. The colony PCR was verified by 2% agarose gel electrophoresis. Correct colony was chosen to isolate the plasmid for sequencing using standard primers of pMiniT 2.0. The sequencing of chosen candidates were performed by Microsynth (Vienna, Austria) and the results were analysed by Benchling software (CA, USA) and were presented in Table S3. Predicted secondary structures of aptamers were analyzed by the Unafold server with the set-up conditions: 25 °C, 157 mM Na^{+} and 0 mM Mg^{2+}.

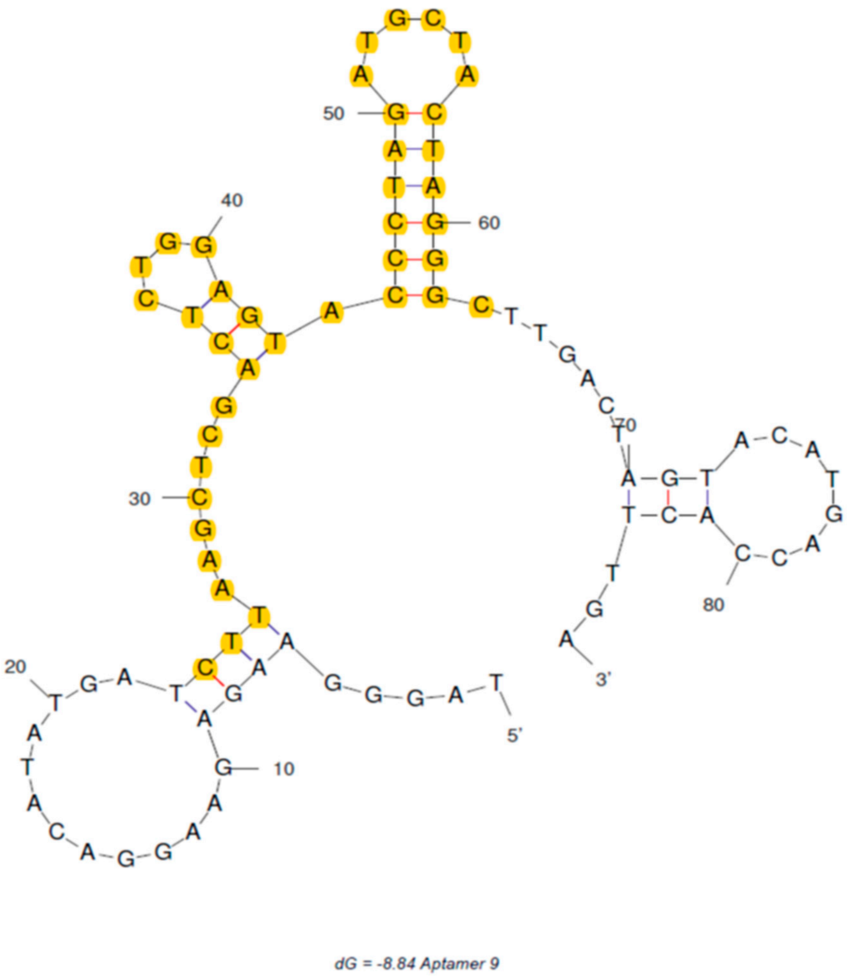

Figure S1: Predicted secondary structrure of the aptamer APS9 by Unafold web server with the set-up conditions: at 25 °C, 157 mM Na^{+} and 0 mM Mg^{2+}. The APS9 possesses one single-stranded segment, 4 hairpin-loops with ΔG = -8.84 kcal/mol. Random oligonucleotides are highlighted.

Table S1: Component of BG110 (Std) in 1L (BCCM, Belgium).

Component	Final conc (mM)
$K_2HPO_4.3H_2O$	0.1752
$MgSO_4.7H_2O$	0.623
$CaCl_2.2H_2O$	0.2448
EDTA-Mg-Na_2	0.0226
Citric acid	0.0286
$C_6H_8FeNO_7$	0.0226
Na_2CO_3	0.2
$NaHCO_3$	1.8
Microelements*	1 ml

* Trace metal mix A5 with Co (Sigma, St. Louis, USA)

Table S2: Component of BG11 medium in 1L (BCCM, Belgium)

Component	Final conc (mM)
$NaNO_3$	17.66
$K_2HPO_4.3H_2O$	0.1752
$MgSO_4.7H_2O$	0.623
$CaCl_2.2H_2O$	0.2448
EDTA-Mg-Na_2	0.0226
Citric acid	0.0286
$C_6H_8FeNO_7$	0.0226
Na_2CO_3	0.2
$NaHCO_3$	1.8
Microelements*	1 ml

* Trace metal mix A5 with Co (Sigma, St. Louis, USA)

Table S3: Sequences of aptamer candidates analyzed by sequencing.

Name	Sequence (5′-3′)
APS2	TAGGGAAGAGAAGGACATATGATTTGTGTG-GACATGAGGTGGGCGGCCGCGGGTGGTTGGGCTTCCCGGTCGGTGGTTGGGTTG
APS3	TAGGGAAGAGAAAGGACATATGATGGGGCTGATAGTGCCTGG-CAATTATTGTGAAAGTTTATTTGACTAGTACATGACCACTTGA
APS4	TAGGGAAGAGAAGGACATATGATTGGGGAGGGCGAGGTTTGCAGAGGGA-GAAAAGGTTGCTGT TTGACTAGTACATGACCACTTGA
APS6	TAAGGGCGACACGGAAATGTTGAATACTCATACTCTTCYTTTTTCAATATTATTGAA-GCATTTATCAGGGTTATTGTCTCATGA
APS8	TAGGGAAGAGAAGGACATATGATATATTGGGACATTTCCGGAGAGTCCAGACATGTT-GGGAGGTTGACTAGTACATGACCACTTGA
APS9	TAGGGAAGAGAAGGACATATGATCTTAAGCTCGACTCTGGAGTACCCTAGATGC-TACTAGGGCTTGACTAGTACATGACCACTTGA
APS10	TAGGGAAGAGAAGGACATATGATATAGACCCAGTGGTACAGCATAGGCTGACTTT-GTTCGCTATTGACTAGTACATGACCACTTGA
APS11	TAGGGAAGAGAAGGACATATGATGAGAGGAAAGTATGATGGGTTTTTGGGTGG-CAAGTCATGTTTGACTAGTACATGACCACTTGA
